# Anatomical stability of human fungiform papillae and relationship with oral perception measured by salivary response and intensity rating

**DOI:** 10.1038/s41598-019-46093-z

**Published:** 2019-07-05

**Authors:** Alexander Gardner, Guy H. Carpenter

**Affiliations:** Salivary Research, Centre for Host-Microbiome Interactions, Faculty of Dentistry, Oral & Craniofacial Sciences, King’s College London, London, UK

**Keywords:** Saliva, Proteins, Neurophysiology

## Abstract

Fungiform papillae house taste buds on the anterior dorsal tongue. Literature is inconclusive as to whether taste perception correlates with fungiform papillae density (FPD). Gustatory reflexes modulate the amount and composition of saliva subsequently produced, and thus may be a more physiologically objective measure of tastant-receptor interactions. Taste perception fluctuates with time but the stability of individual fungiform papillae is unclear. This study followed ten healthy volunteers longitudinally at baseline, one and six months. FPD, diameter and position were measured and participants rated intensity perception of sucrose, caffeine, menthol and capsaicin solutions. Salivary flow rate, protein concentration and relative changes in protein composition were measured following each tastant. FPD, diameter and position were unchanged at six months. FPD did not correlate with intensity rating for any taste. FPD did correlate with changes in salivary protein output following sucrose (ρ = 0.72, p = 0.02) and changes in levels of proline-rich protein and mucin 7 following capsaicin (ρ = 0.71, p = 0.02, ρ = 0.68, p = 0.04, respectively). These results suggest that over six months fungiform papillae are anatomically stable, playing a greater role in mediating the physiological salivary response to stimuli rather than determining the perceived intensity of taste.

## Introduction

Fungiform (mushroom shaped) papillae are small projections located on the anterior two thirds of the dorsum of the tongue, housing collections of chemosensory cells (taste buds) and nerves^1^. Fungiform papillae density (FPD) is invariably reported to vary widely between individuals, from 0 cm^−2^ to >200 cm^−2^, although mean density approximates 100 cm^−2^ ^2,3^. Importantly, it has been shown that FPD is not uniform across the dorsum of the tongue, with a higher density occurring anteriorly and medially^4,5^. As with papillae density, the density of taste pores (the pore connecting taste buds with the oral cavity, used as an indicator of the presence of a taste bud)^6^, varies considerably. One papilla may house up to 22 taste pores with taste pore density ranging from 36 to 511 pores cm^−2^ ^7^.

FPD changes throughout life, reducing relatively rapidly during early development between ages 7 and 10, coinciding with tongue development^4^. Morphological differences are also recognised with children having rounder papillae than adults^5^. During maturity there is evidence of a more gradual decline of 2.8 papillae cm^−2^ with every five years of age^3^. This data suggests that following early development, papillae density is relatively stable. However, these measurements are based on means from samples of individuals classified by age. It would therefore be conceivable that at an individual level there may be fluctuations in FPD that are masked at a population level. Individual taste perceptions have been shown to fluctuate with time, so should papillae density also fluctuate this may help explain these observations^8^.

A study of taste bud volume within fungiform papillae indicated that papillae diameter fluctuates week to week^9^, however, no literature following participants longitudinally to investigate stability of their fungiform papillae exists. While it is known that the turnover of taste bud cells is approximately 10 days^10^, it is unknown whether fungiform papillae themselves undergo turnover or migration at a macroscopic level.

Multiple studies have looked at measuring FPD as a marker of taste sensitivity, investigating the hypothesis that individuals with a higher FPD may therefore have a greater perception of intensity. Results have been conflicting, likely due to variability in protocol and difficulty in rating individual taste perception. It was originally observed that intensity perception of 6-*n*-propylthiouracil (PROP), which is perceived as bitter at considerably lower concentrations depending on polymorphisms in the bitter receptor gene *TAS2R38*^11^, correlated with FPD^12,13^. Higher FPD has also been related to increased perception of sucrose, sodium chloride and citric acid in multiple studies^2,14–17^. More recently, epidemiological data (n = 2371) found no relationship between FPD and intensity rating for sucrose, quinine, sodium chloride citric acid or PROP^3^. There is further strong evidence that there is no relationship between PROP bitterness and FPD^18^.

Fungiform papillae density in relationship to other oral perceptions is less studied than for the basic tastes. There is evidence that fungiform papillae play a role in oral proprioception and perception of transient receptor potential (TRP) agonists, such as the perceived intensity of the oral sensation of ethanol^19^. The detection of creaminess has related to FPD, suggesting a role of papillae in the oral perception of fat^20^. While tactile sensitivity of the anterior tongue correlates with FPD and papillae diameter in an objective task^21^, such a relationship does not necessarily translate to perception of food roughness^22^.

There are numerous protocol considerations that may account for the variability in literature regarding oral perception and FPD. These include lack of a standardised region of the tongue being measured and difficulty identifying fungiform papillae. Measuring FPD requires training and where multiple operators are measuring FPD inter-operator consistency should be performed^3,23^. Other variables include concentration and route of administration of tastant. Tastant administration methods include application of tastant impregnated filter discs^24^, applying tastant via cotton buds^14^, having participants hold tastant solution in the mouth^2^, and use of customised application devices^16,17^. Concentrations of tastants vary considerably, ranging from 0.21 M to 1.8 M sucrose and 5.5 mM to 1 M PROP^3,15,24^. Excessively low or high levels of tastant appear to contribute to aberrant results as evidenced by PROP intensity failing to correlate with papillae density when applied by overly small or large filter discs, but correlating with intermediately sized discs^24^. Finally, assessment of taste perception is varied. While some studies measure taste acuity by determining detection thresholds^2,16^, more commonly perception intensity is rated on a scale. The labelled magnitude scale is commonly used^3,21^, however others such as the nine-point scale have been used^14^. The former scale has been identified as being superior to the latter^8^, whereas others caution its use in sensory research^25^.

An alternative method of assessing taste, which to our knowledge has not been investigated with respect to FPD, is the assessment of salivary response. Taste perception has long been recognised as an important physiological stimulus of reflex salivary production^26^. Following tastant binding at receptors, sensory information is relayed to the salivary glands via nuclei in the brainstem in an autonomic process^27^. This is true not only for the volume of saliva produced, which increases more markedly for sour tastes, followed by salt and then sweet and bitter^28^, but the salivary composition is also affected^29^. Dawes *et al*. found salt had a significant effect on salivary protein content and calcium concentration, whereas the effects of other basic tastants were generally comparable^30^. More recently, mass spectroscopy has been utilised to show salivary protein changes were most pronounced following sour stimulation, followed by bitter, umami, then sweet^31^. Due to the autonomic physiological relationship between taste perception and saliva production, analysis of salivary response following taste removes both the subjectivity of asking participants to rate their perception and the methodological complexity and inherent inaccuracy of determining taste thresholds. This study aimed to evaluate the anatomical stability of fungiform papillae by measuring papillae density, diameter and relative position of participants followed longitudinally at one and 6 months from baseline. The second aim was to compare the relationship between FPD and salivary response to taste with the relationship between FPD and oral perception measured by self-reported intensity ratings.

## Results

### Fungiform papillae density, diameter and position is stable over six months observation

**Figure 1:**
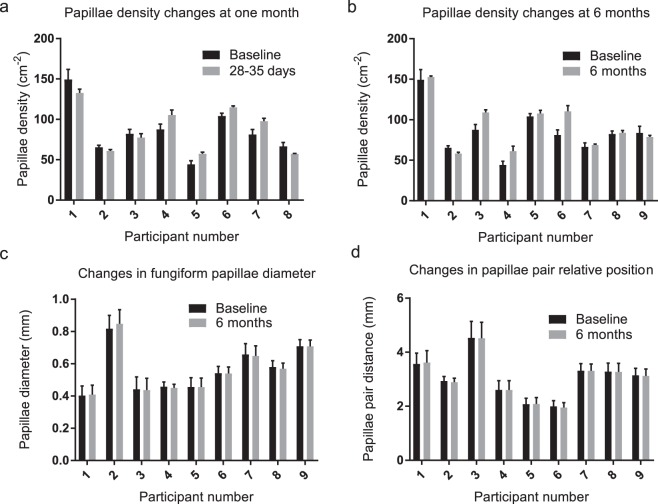
Mean papillae density (±s.e.m.) based on three counts of the same image by a single operator at one month (3**a**) and six months (3**b**) from baseline. The different participant numbers are due to participant drop-out. No significant differences in papillae density were observed at one or six months (Wilcoxon signed-rank test, p = 0.54 and 0.20, respectively). (**c**,**d**) Show data for papillae diameter and relative position at baseline and six months for nine individuals, (n = 10 measures per participant at each time point). Two-tailed paired t-test revealed no significant differences in diameter (p-value range 0.32–0.95) or papillae position (p-value range 0.15–0.94).

**Figure 2:**
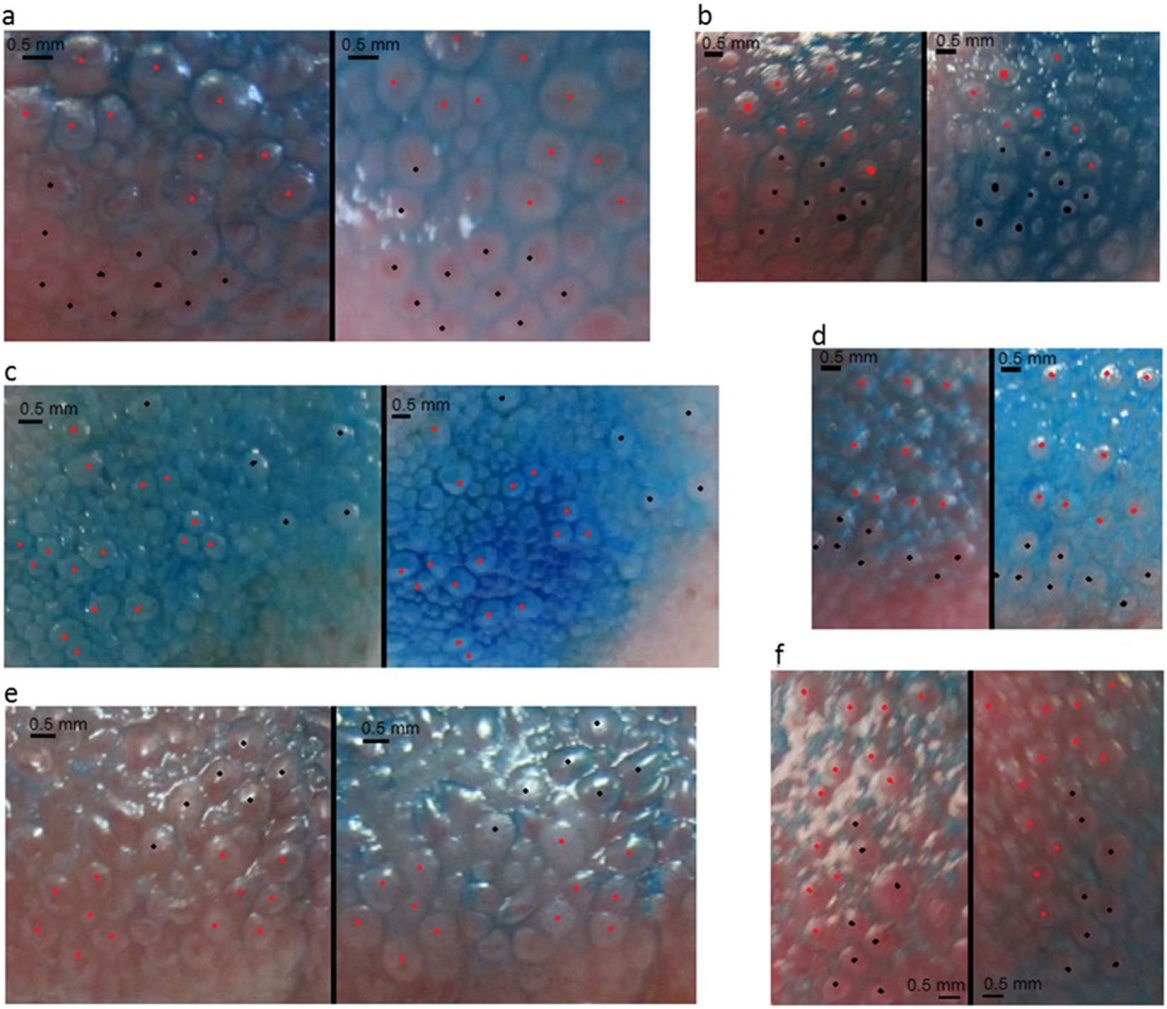
Selection of participant photographs at baseline (left) and 6 months (right). Distinctively shaped clusters of papillae have been identified at both time points and marked with red and black dots to aid visualisation. The different colours are purely to aid visualisation. No manipulations of photographs have been made apart from cropping.

Fungiform papillae density was measured in thirteen individuals (M = 6, F = 7) with a mean of 88 and range of 44–149 papillae/cm^2^. Inspection for normality suggested a right-skewed distribution. Eight participants returned at one month (28–35 days) and nine returned at 6 months (180–188 days). Papillae density comparisons are shown in Fig. 1a,b. Wilcoxon signed rank test revealed no significant differences in papillae density at one or six months (p = 0.54, 0.20) respectively. Although some fluctuation in FPD can be observed at different time points this variability was no greater than intra-operator measurement variability. Average papillae diameter ranged from 0.40 to 0.85 mm, across participants the mean was 0.56 mm. There was no significant change in fungiform papillae diameter for any participant between baseline and six months, Fig. 1c. Given no measurable differences were detected over after six months, measurements at one month were not performed. There were no significant differences found in the average distance between paired papillae between six months and baseline for any participant, Fig. 1d. The anatomical stability of fungiform papillae is shown in Fig. 2, revealing clusters of distinct papillae at baseline and six months in several participants.

### Capsaicin, menthol and sucrose increase salivary flow rate without changing total protein concentration

At the initial visit participants scored tastant intensity in the correct order of concentration in every case but one, where one participant confused the higher two sucrose concentrations. Choice of tastants for subsequent anchored assessment was based on the spread of initial participant ratings, shown in Supplementary Fig. 1. A tendency for clustering of ratings for excessively low or high concentrations can be seen, e.g. 1 M sucrose and 0.1 ppm capsaicin. Tastant concentrations selected for definitive rating were 0.25 M sucrose, 8 mM caffeine, 1 ppm capsaicin based on the symmetric distribution of responses, mean responses approximating the midpoint of the scale and the highest variance suggesting optimal discriminatory ability of participant taste sensitivity. For menthol, 250 ppm was selected. The mean rating was slightly higher than the scale midpoint compared ratings for 100 ppm menthol however the ratings for 100 ppm menthol appeared negatively skewed, due to an outlying high rating (Supplementary Fig. 1).

**Figure 3:**
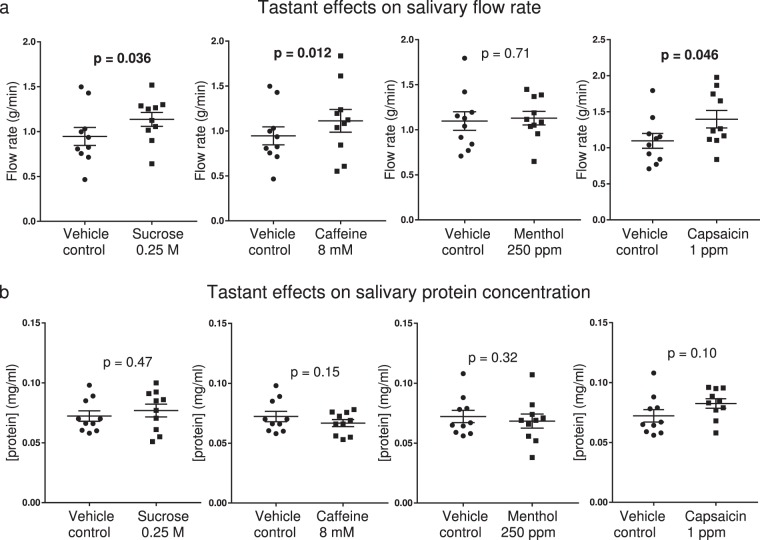
Effects of tastants on salivary flow rate and protein concentration (bars represent mean ± s.e.m., n = 10). P-values shown are for two-tailed paired t-tests, values in bold reflect significance (p < 0.05). Vehicle controls were either water when comparing caffeine or sucrose, or 0.475% ethanol in water when comparing menthol or capsaicin. (**a**) Sucrose, caffeine and capsaicin increased flow rate significantly vehicle control solutions. (**b**) Protein concentration was not significantly different from vehicle control for any tastant rinse.

Flow rate was not significantly increased above unstimulated levels by vehicle control rinses. Significant increases relative to vehicle control were detected following sucrose, caffeine and capsaicin solutions (p = 0.036, 0.012, 0.046 respectively), Fig. 3a. Protein concentration was not significantly changed relative to vehicle controls for any tastant solution, Fig. 3b.

### Capsaicin stimulation increases the relative abundance of several major salivary proteins

**Figure 4:**
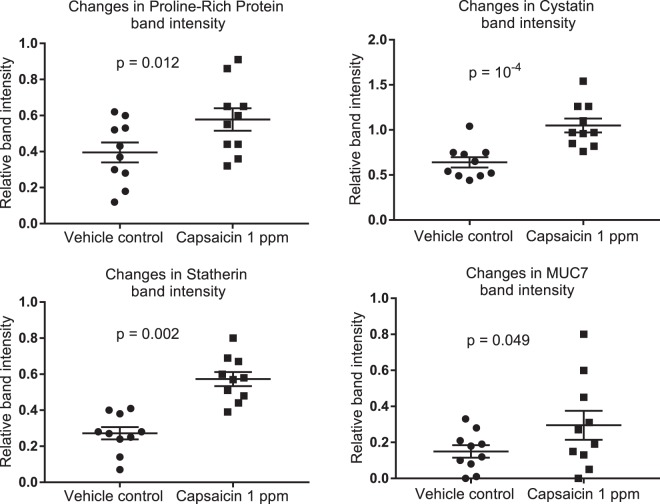
Mean ± s.e.m. band intensity changes between capsaicin 1 ppm rinses and 0.475% ethanol vehicle control rinses. p-values shown reflect two-tailed paired t-test, n = 10. No significant changes were detected for other tastants. Band intensity, as measured by SDS-PAGE densitometry, is expressed relative to standard reference samples run on every gel.

Analysis of changes in relative intensity of individual protein bands indicated that capsaicin-stimulated saliva showed statistically higher band intensity for cystatin, statherin, MUC7 and proline-rich protein bands when compared to the ethanol vehicle control stimulated saliva, Fig. 4. No significant differences were found between tastants and vehicle controls for sucrose, caffeine or menthol. No significant difference between any tastant and vehicle control were found for amylase or MUC5B.

### Papillae density correlates with tastant stimulated changes in saliva composition

**Figure 5:**
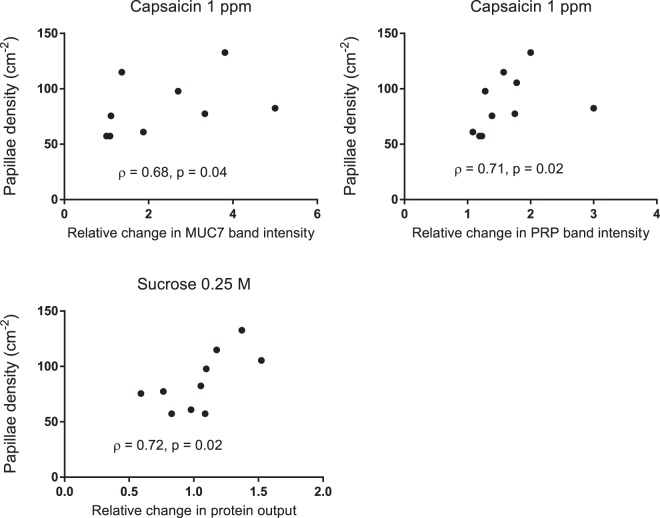
Correlations between FPD and salivary changes following capsaicin 1ppm and sucrose 0.25 M. Although a somewhat linear relationship appears to exist no lines have been fitted due to the use of Spearman’s rank correlation (n = 10).

No correlation was found between intensity ratings and FPD for any tastant solution. Significant correlations were found between FPD and salivary changes for MUC7 and Proline-rich protein band intensity following 1 ppm capsaicin (ρ = 0.68, p = 0.04, ρ = 0.71 p < 0.02, respectively), Fig. 5. FPD was also found to correlate with changes in salivary protein output following sucrose 0.25 M (ρ = 0.72, p = 0.02), Fig. 5.

## Discussion

This study intended to assess the anatomical stability of fungiform papillae over six months. Our results indicate fungiform papillae are essentially stable surface features on the tongue, with no change in papillae density, diameter, or position at least over a six-month time course in healthy individuals. This knowledge could be useful, for example, investigation into the pathogenesis of conditions involving oral dysesthesia such as burning mouth syndrome, in which FPD has been implicated^32^. Longitudinal studies of taste perception, where wide daily fluctuation in sensory response to the same stimuli can occur^8^, may also benefit from removing FPD as a variable contributing in the day-to-day variation in taste assessment. Although papillae themselves appear stable, taste buds within papillae may change in volume as taste receptor cells undergo turnover^9^. Whether this could transiently influence perception is unclear as, in health, taste bud turnover is thought to be a homeostatic process rather than displaying temporal net increase or reduction in taste receptor cells^10^.

A further aim was to investigate the relationship between FPD and both taste perception and taste stimulated salivary reflexes. Despite epidemiological evidence suggesting FPD does not relate to taste perception^3^, FPD remains a commonly used phenotype in taste research, although results continue to be inconclusive^33,34^. Sucrose and caffeine are common tastants representing sweet and bitter basic taste modalities, used in clinical taste assessment^35,36^. Capsaicin and menthol are agonists of transient receptor potential channels, conferring their respective warming and cooling sensations via channels TRPv1 (capsaicin) and TRPm8 (menthol). Sucrose and caffeine perception have previously been studied relating to FPD^2,33^, and while capsaicin has not been directly studied with respect to FPD, perception of ethanol (another TRPv1 agonist), has been^37^. No significant association between FPD and taste perception was detected in this study.

Salivary responses to TRP agonists are less studied than those of basic tastes. Our results showed that 0.25 M sucrose, 8 mM caffeine, and 1 ppm capsaicin significantly increased salivary flow rate above that stimulated by vehicle controls. Sucrose has been shown to stimulate salivary flow of whole mouth and parotid saliva, although effects are short lived in the order of minutes^38,39^. Capsaicin has also been shown to increase salivary flow, with greater effects being seen on the submandibular and sublingual glands, sustained for around five minutes, albeit at concentrations of 9 ppm^40^. Nasrawi *et al*. divided participants into “high” (flow rate >100 mg min^−1^) and “low” flow individuals based on their unstimulated flow rate and found capsaicin only significantly stimulated salivary flow in high flow rate individuals. Whether this approach is founded in a genuine physiological difference between individuals is unclear.

Regarding salivary protein changes, no significant changes were found for sucrose, caffeine or menthol. While mass-spectroscopy based studies have found selected salivary protein changes following sweet stimuli these effects appear to be minimal and short-lived and would not likely be picked up gel electrophoresis^31,41^. Our results found that 1 ppm capsaicin was the only tastant to significantly increase protein band intensity and did so for several proteins. Such an effect has not been reported in literature, however there is evidence in rats that capsaicin will induce cystatin-s like protein production in the submandibular glands^42^. The salivary proteins observed to be increased by capsaicin stimulation were cystatin, statherin, proline-rich protein and MUC7. Cystatin and MUC7 are produced by the submandibular and sublingual glands. Cystatin is a cysteine protease inhibitor whereas MUC7 is a glycoprotein contributing to the viscoelasticity of saliva^29,43^. Proline-rich proteins are largely produced by the parotid gland and along with statherin are involved in pellicle formation. Statherin is a calcium binding protein and its concentration reduces upon entering the mouth due to proteolysis and tissue binding^29,44^, thus can indicate a rapidly produced, stimulated saliva. These observations taken together suggest capsaicin caused a rapid increased protein output from all major salivary glands.

In contrast to sensory responses, several of the observed salivary changes correlated positively with FPD. These were for proline-rich protein and MUC7 increases following 1 ppm capsaicin and protein output following 0.25 M sucrose. It therefore appears that these stimulatory effects of capsaicin are enhanced in individuals with higher FPD. These associations may reflect the objective nature of measuring saliva compared to complexities of measuring participant intensity perception which can be influenced by more complex neural inputs such as by hedonic response, memory and expectation^45,46^. An explanation for why capsaicin stimulated changes were found to have the strongest association with FPD may lie in the fact TRPv1 nerve fibres have been shown to enter human fungiform papillae although are absent from surrounding epithelium^47^. Furthermore, fMRI imaging has shown capsaicin activates autonomic brain regions (anterior short gyrus of the insula) to a significantly greater extent than gustatory centres (middle and posterior short gyri of the insula)^48,49^. Therefore, TRPv1 expressing neurones within fungiform papillae could be relaying autonomic rather than sensory responses to the brain, initiating subsequent salivary reflexes.

This study aimed to investigate the stability of fungiform papillae in the same individuals over six months. Correlation between FPD and taste perception measured by both participant ratings of intensity and physiological changes in salivary flow and composition were also investigated. Fungiform papillae did not change in density, size or position over a six-month period. FPD did not correlate with intensity ratings, however significant correlations between salivary changes were observed for protein output following sucrose and MUC7 and proline-rich protein following capsaicin. To our knowledge FPD and taste-stimulated salivary changes have not been studied previously. While this work does not prove definitively that FPD is proportionate to the magnitude of salivary reflexes, it does suggest fungiform papillae mediate the physiological salivary response to capsaicin more so than perceived intensity of the stimulus. This concept could be explored further in the fields of sensory science and oral pathology.

## Methods

### Participant recruitment and study design

All research was conducted in accordance with the Declaration of Helsinki, following ethical approval from King’s College London ethics committee (HR-15/16–2508). Participants were invited to volunteer and then screened for compliance against the following exclusion criteria: no diagnosed disorders of taste, no acute oral conditions and no objection to the tastants. Written informed consent was obtained. Thirteen healthy volunteers (six male and seven female) participated, aged 23 to 42 at the time of commencing the study.

Volunteers were assessed on three occasions. At the initial visit participants were familiarised with the tastants, rated an initial panel of tastants to determine appropriate concentrations (i.e. above recognition threshold but avoiding ceiling effects) and their tongues were photographed. One month subsequently, participants were asked to rate their perception of tastants. At this second visit, saliva was collected for biochemical analysis at baseline and after each tastant. Flow rates were recorded, and the tongue was photographed. At the third and final visit, timed six months from the initial visit, the tongue was photographed a third time. Data collected at each visit was treated as distinct and not averaged between visits.

### Tastant preparation, administration and participant intensity rating

Sucrose, caffeine, menthol and capsaicin were purchased from Sigma-Aldrich at either food-grade or pharmaceutical-grade rating. Food-grade ethanol was purchased to assist in solubilising the latter two tastants. Bottled water (Buxton) was used as a standardised solvent source. Tastants evaluated at visit one for purposes of concentration selection are summarised in Table 1.

**Table 1:** Summary of tastant and solvent concentrations investigated at the initial visit. All concentrations in ppm and percentage are by volume.

Tastant	Taste/oral sensation	Concentrations investigated	Solvent
Sucrose	Sweet	0.25 M, 0.5 M, 1 M	Water
Caffeine	Bitter	4 mM, 8 mM, 20 mM	Water
L-Menthol, (1 *R*,2 *S*,5 *R* stereoisomer)	Cooling sensation	50 ppm, 100 ppm, 250 ppm	Water and ethanol (9.5 × 10^−3^%, 0.019% and 0.475% respectively)
Capsaicin	Warming sensation	0.1 ppm, 1 ppm, 2.5 ppm	Water and ethanol (9.5 × 10^−3^%, 0.095% and 0.24% respectively)

All taste assessment was performed at room temperature of 19 °C. Participants were instructed to avoid eating, drinking, smoking and chewing gum for one hour before tasting. Tastants were presented at different concentrations in 10 ml volumes. Participants were seated and asked to hold the solution into the mouth, not rinsing or swallowing, pooling the liquid at the floor of the mouth. Tastants were expectorated after 30 seconds and ratings were recorded. Participants were asked to inform the researcher when the previous taste was no longer present in the mouth before moving to the next tastant, usually after several minutes. The order of tastant administration was standardised across participants (sucrose, caffeine, menthol then capsaicin) since capsaicin had a long lasting oral effect. Where multiple concentrations of the same tastant were investigated, participants were blinded to the concentration and order of the different concentrations. Ratings were recorded on a 0–10 graphic rating scale labelled with both verbal descriptors and numerical graduations, Supplementary Fig. 2. Participants were blinded to the tastant concentrations.

Tastant concentrations for intensity rating at the second visit were selected based on the results of the first visit. Concentrations were 0.25 M sucrose, 8 mM caffeine, 250 ppm menthol and 1 ppm capsaicin. Tasting was performed as described for the initial visit, however for purposes of standardisation both ends of the scale were anchored. The low end anchor was essentially the solvent in the absence of tastant, the high end anchor represented a higher concentration of the same tastant solution. The tastant and anchor solution concentrations are summarised in Supplementary Table 1.

### Saliva collection and analysis

Following expectoration of tastant, saliva was collected into pre-weighed universal tubes over a period of two minutes. An initial unstimulated sample was obtained, followed by samples subsequent to water and ethanol vehicle controls (the zero anchors for the rating scale) and the four tastants, giving a total of seven samples per participant. Tubes were re-weighed and flow rate was calculated in g/min.

Samples were kept on ice during collection and centrifuged at 4600 *g* for ten minutes and supernatant was rapidly frozen and stored at −80 °C. Supernatant was assayed for total protein content (BCA assay, Thermo Scientific, Rockford, IL, USA). Protein output was calculated as the product of flow rate and total protein concentration.

Individual proteins were analysed by 1-dimensional sodium dodecyl sulphate polyacrylamide gel electrophoresis (1D-SDSPAGE). 15 µl supernatant was mixed with 5 µl sample buffer (NuPAGE LDS sample buffer, Thermo Fisher Scientific, Carlsbad, CA, USA) and 2 µl 0.5 M dithiothreitol (DTT, Sigma-Aldrich, St. Louis, MO, USA). Samples were vortexed, heated at 100 °C for 3 mins then centrifuged at 4600 *g* for 3 mins and 10 µl of buffered sample was loaded into pre-cast polyacrylamide gels (NuPAGE 4–12% Bis-Tris Gel, Thermo Fisher Scientific, Carlsbad, CA, USA) and electrophoresed at 200 V, 250 mA, for 32 mins in a 5% running buffer solution (NuPAGE MES SDS running buffer, Thermo Fisher Scientific, Carlsbad, CA, USA).

To compare protein staining between gels every gel contained one lane with a sample of pre-aliquoted reference saliva prepared in the same way. Following electrophoresis, gels were stained with Coomassie brilliant blue (Sigma- Aldrich, St. Louis, MO, USA) diluted 2:3 with acetic acid for 30 minutes. Gels were destained with 10% acetic acid and imaged using a ChemiDoc^TM^ MP imaging system and analysed in ImageLab 5.2.1 (Bio-Rad Laboratories, Hercules, CA, USA). Lanes and bands were automatically detected, corrected manually and band intensity peaks were referenced to the cystatin band on the reference saliva lane, which was run on every gel. A uniform background filter (equivalent to the signal of an empty lane) was applied to every lane. Protein band assignment was conducted as described previously^29^, using the molecular weight marker as a guide. Where proline-rich protein is mentioned this describes the prominent band around 30 kDa.

Gels were then stained for glycoproteins with periodic acid Schiff’s stain (PAS). Gels were stained in 2% periodic acid (Sigma-Aldrich, St. Louis, MO, USA) for 20 minutes, washed three times in Milipore filtered water for 5 minutes then stained with Schiff’s reagent (VWR) for thirty minutes in a light-proof tray. Gels were destained in UHQ water, imaged and analysed as for coomassie stained gels using the MUC5B band of the reference saliva lane to reference peak intensity, as previously described^43^. One participant was excluded from analysis of MUC7 as the band was not detected in any of their salivary samples. Supplementary Fig. 3 illustrates a typical stained gel showing the protein bands analysed.

### Fungiform papillae measurements

**Figure 6:**
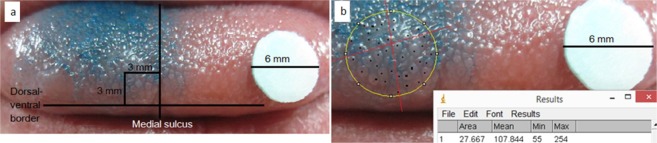
Illustration of fungiform papillae counting method. (**a**) Outlines the process of selecting a standardised point to centre a 3 mm radius circular area. (**b**) Shows the process of calibrating this area relative to the 6 mm diameter disc in ImageJ and counting papillae.

The anterior dorsal surface of the tongue was stained with one drop of Brilliant Blue FCF food dye (PME, Enfield, UK). Due to the potential for the dye to affect taste and salivary chemistry this step was conducted after tasting and saliva collection. A sterilised 6 mm diameter filter disc was placed on the tongue for calibration purposes and a digital photograph was taken. Images were analysed in ImageJ (NIH, USA). To ensure a standardised tongue region was measured a circular area approximately 6 mm in diameter was measured centred around a point 3 mm lateral to the medial lingual sulcus and 3 mm posterior to the non-keratinised mucosal border of the tongue (Fig. 6a). The diameter of the circular area was measured, the papillae within the circle were counted and a density of papillae/cm^2^ was calculated (Fig. 6b). For every image this process was conducted by a single operator and repeated on three non-consecutive days without reference to previous counts to establish a mean papillae density.

To investigate changes in papillae size and position diameter was recorded for the ten papillae closest to the central point at baseline and the same papillae were identified 6 months later and re-measured. As many papillae are not perfectly circular, the greatest dimension was measured as opposed to a true diameter. Additionally, pairs of papillae identified at baseline and six months were identified and their relative separation measures, Supplementary Fig. 4. The same papillae were identified by their morphology at both time points. Measurements between the same five papillae pairs in the antero-posterior direction and five pairs medial-laterally were performed at both times points and differences were assessed by two-tailed paired t-test.

### Statistical analysis

All data were analysed in SPSS 24 (IBM). Data were inspected and tested for normality by Q-Q plots and Shapiro-Wilk test and appropriate statistical tests selected. P-values below 0.05 were considered to be significant. Changes in FPD were assessed by Wilcoxon-signed rank test. Papillae diameter and papillae position were measured by two-tailed paired t-test. All salivary data (flow rate, total protein and protein band intensity) were analysed by repeated-measures ANOVA with Greenhouse–Geisser correction of sphericity and Bonferroni post-hoc tests to account for multiple comparisons. Having satisfied the ANOVA, comparisons between tastant and vehicle control bands and analysed by two-tailed paired t-test. For correlation analyses Spearman’s rank correlation was used given FPD was non-parametric.

## Supplementary information

Supplementary Material

## Data Availability

Gel scans and images are available from the corresponding author on reasonable request.
