# Supplementary material for: Anatomical stability of human fungiform papillae and relationship with oral perception measured by salivary response and intensity rating
Source: Sci Rep. 2019 Jul 5;9:9759. doi: 10.1038/s41598-019-46093-z (PMC6611804; doi:10.1038/s41598-019-46093-z)
Supplement: Supplementary file 1 — Supplementary Material [file 41598_2019_46093_MOESM1_ESM.pdf]

Anatomical stability of human fungiform papillae and relationship with oral perception measured by salivary response and intensity rating

Alexander Gardner[1,*], Guy H. Carpenter[1]
1. Department of Mucosal and Salivary Biology, Dental Institute, King's College London
***Corresponding author: alexander.gardner@kcl.ac.uk**

Supplementary material

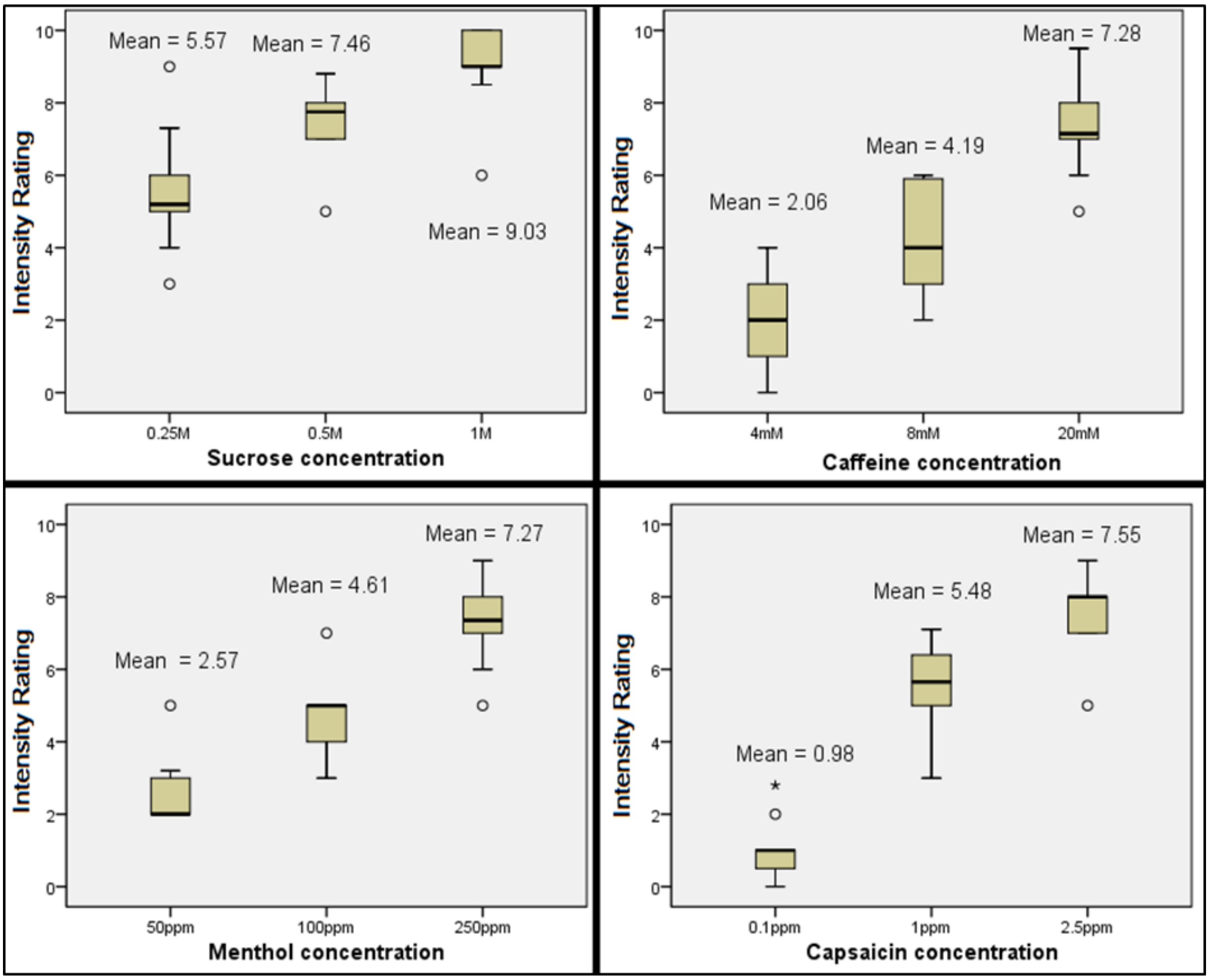

Supplementary Figure 1: Boxplots illustrating the spread of participant scores for the panel of tastant concentrations rated at the first visit, (n=10).

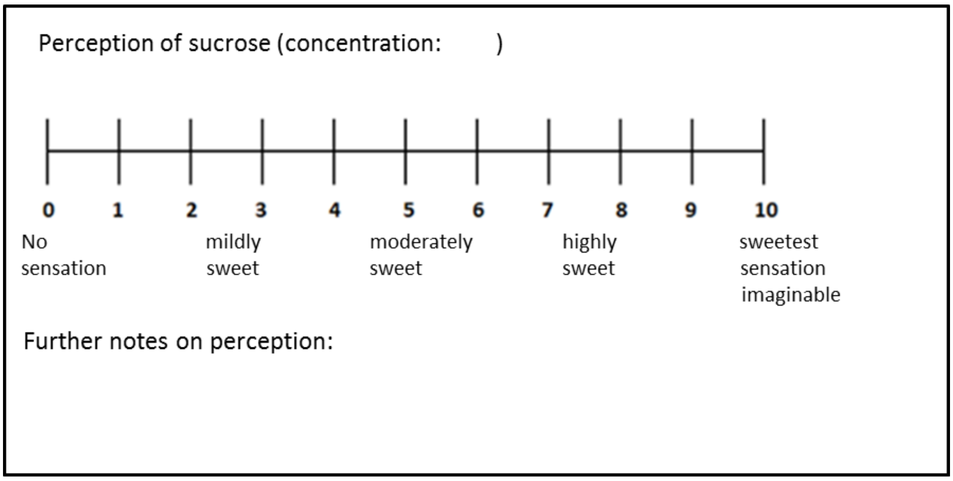

Supplementary Figure 2: Example graphic rating scale used for participants intensity ratings. Concentration was completed following the participants rating to maintain participant blinding.

**Tastant concentration rated**	**"Zero" anchor solution**	**"Ten" anchor solution**
Sucrose 0.25 M	Water	Sucrose 1 M
Caffeine 8 mM	Water	Caffeine 20 mM
Menthol 250 ppm	Ethanol 0.475 %	Menthol 500 ppm
Capsaicin 1 ppm	Ethanol 0.475 %	Capsaicin 2.5 ppm

Supplementary table 1: Summary of tastant concentrations and low and high anchor solutions rated by participants at the second visit.

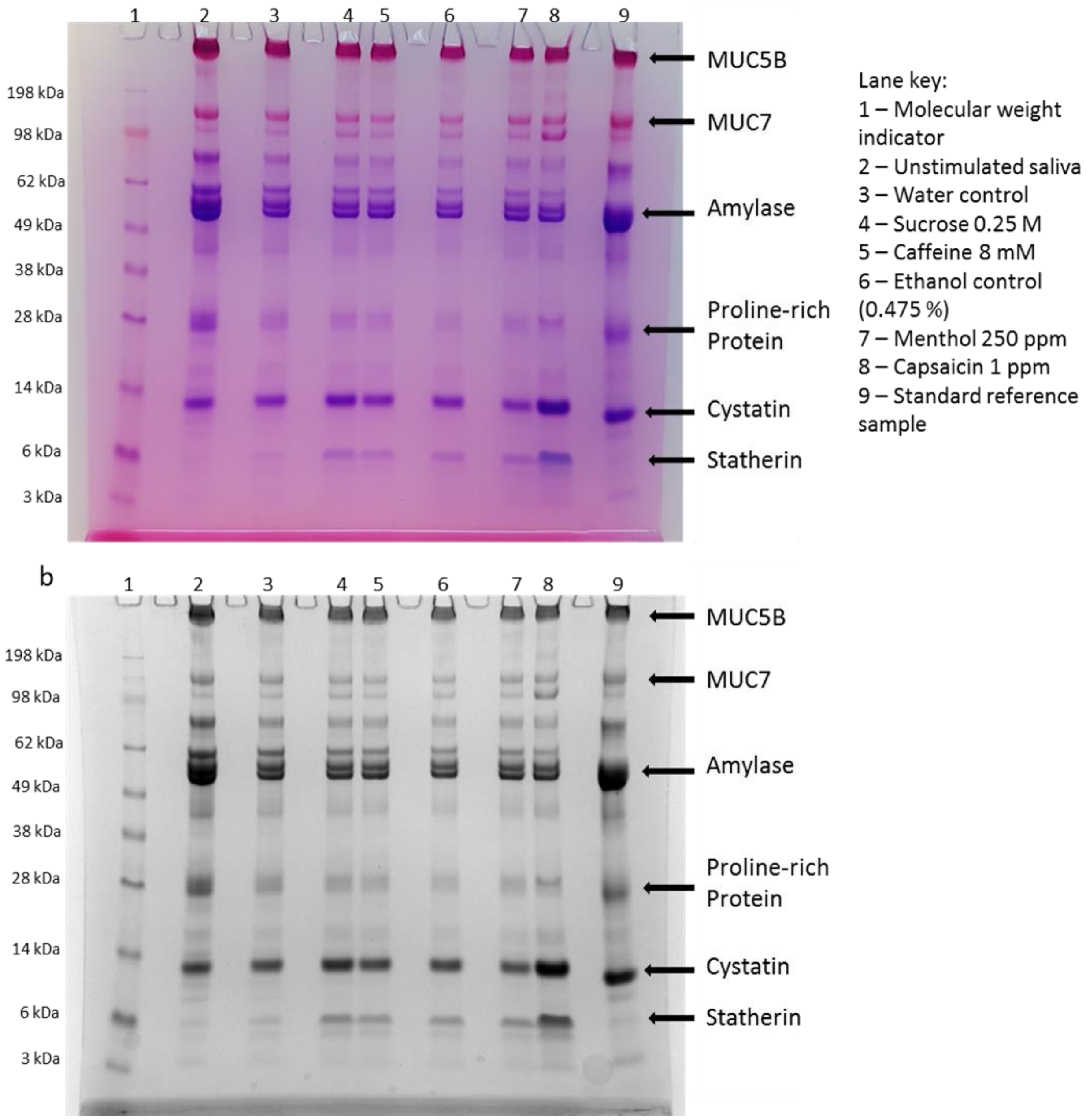

Supplementary Figure 3: Photograph (3a) and Chemidoc scan (3b) of the same coomassie and PAS stained polyacrylamide gel, illustrating sample lay out. Saliva sample stimuli are identified above the lanes, molecular weight markers on the left and the bands of interest are identified on the right. MUC5B = mucin 5B, MUC7 = mucin 7. Each lane is loaded with an equal volume (10µl) of buffered sample. Photograph (3a) was taken under natural light with no flash. Chemidoc image (3b) was acquired under Coomassie protein gel settings with automatic exposure set for intense bands. No digital manipulations have been made to images post acquisition.

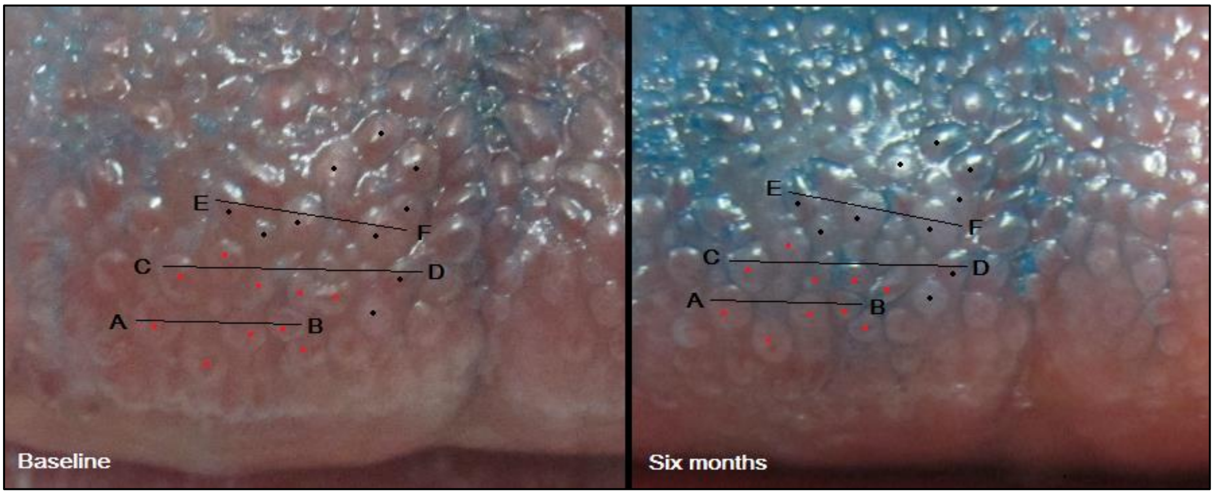

Supplementary Figure 4: Example of measurement of relative papillae position six months apart. Clusters of papillae are identified at both time points and labelled by red and black dots. The distance between ten pairs of papillae was measured (five medial-lateral and five anterior-posterior). Only three medial-lateral pairs are marked for purposes of illustration.
